# The canonical equation of adaptive dynamics for life histories: from fitness-returns to selection gradients and Pontryagin’s maximum principle

**DOI:** 10.1007/s00285-015-0938-4

**Published:** 2015-11-19

**Authors:** Johan A. Jacob Metz, Kateřina Staňková, Jacob Johansson

**Affiliations:** Mathematical Institute and Institute of Biology, Leiden University, 2333 CA Leiden, The Netherlands; Evolution and Ecology Program, International Institute for Applied Systems Analysis, 2361 Laxenburg, Austria; Department of Marine Zoology, Naturalis Biodiversity Center, 2333 CR Leiden, The Netherlands; Department of Knowledge Engineering, Maastricht University, 6211 LH Maastricht, The Netherlands; Delft Institute of Applied Mathematics, Delft University of Technology, 2628 CD Delft, The Netherlands; Theoretical Population Ecology and Evolution Group, Department of Biology, Lund University, 22362 Lund, Sweden

**Keywords:** Canonical equation of adaptive dynamics, Function valued traits, Pontryagin’s maximum principle, Age-dependent resource allocation, Mendelian take on life history theory, Evolution in periodic environments, 92D15, 92D40, 37N25, 49K15, 49N90

## Abstract

This paper should be read as addendum to Dieckmann et al. (J Theor Biol 241:370–389, 2006) and Parvinen et al. (J Math Biol 67: 509–533, 2013). Our goal is, using little more than high-school calculus, to (1) exhibit the form of the canonical equation of adaptive dynamics for classical life history problems, where the examples in Dieckmann et al. (J Theor Biol 241:370–389, 2006) and Parvinen et al. (J Math Biol 67: 509–533, 2013) are chosen such that they avoid a number of the problems that one gets in this most relevant of applications, (2) derive the fitness gradient occurring in the CE from simple fitness return arguments, (3) show explicitly that setting said fitness gradient equal to zero results in the classical marginal value principle from evolutionary ecology, (4) show that the latter in turn is equivalent to Pontryagin’s maximum principle, a well known equivalence that however in the literature is given either ex cathedra or is proven with more advanced tools, (5) connect the classical optimisation arguments of life history theory a little better to real biology (Mendelian populations with separate sexes subject to an environmental feedback loop), (6) make a minor improvement to the form of the CE for the examples in Dieckmann et al. and Parvinen et al.

## Introduction

In their recent paper “Function-valued adaptive dynamics and optimal control theory”, Parvinen et al. (2013) give (i) an abstract recipe for calculating the selection gradient for function valued traits affecting the i(ndividual)-dynamics of physiologically structured populations for use in the canonical equation of adaptive dynamics (in the terminology of Metz and Diekmann (1986); Parvinen et al. refer to these models as process-mediated) and (ii) a recipe for calculating the corresponding evolutionarily steady strategies (ESS-es) by using Pontryagin’s maximum principle (c.q. evolutionarily singular strategies (ess-es) if we confine ourselves to the first order condition derived from this principle). They subsequently apply these recipes to derive concrete expressions for three sample models. However, they do not explicitly consider the relationship between (i) and (ii) but for numerically demonstrating that for their special models the adaptive trajectories approach the ess. In this note we (i) demonstrate how the selection gradient can be calculated from a concrete starting point by using the idea of fitness returns, which gives an interpretation to the components of the resulting formulas, and (ii) show that setting the selection gradient equal to zero leads to a classical marginal value argument which turns out to be equivalent to the local version of Pontryagin’s maximum principle.

*Terminology* We employ the term fitness return here for a concept that is widely used in evolutionary ecology, often also under this name, but for which we failed to find an explicit definition. If some fitness proxy can be decomposed as the sum of a number of terms that supposedly stand for the contributions of different pathways by which fitness can accrue, we call the effect of a strategy change on the contribution of a pathway the fitness return through that pathway. For a global ESS the sum of all fitness returns is non-positive whatever the strategy change. For local ESS-es we consider only the fitness returns of infinitesimal strategy changes. To accord with common usage these should be called marginal returns. However, as these are the only returns that we consider we shall drop the epithet. When the attention is confined to an infinitesimal neighbourhood of a reference strategy far more fitness proxies allow a conceptually useful additive decomposition thanks to the rules of differential calculus. All that is needed is a biologically interpretable way in which the proxy can be decomposed as a differentiable function of a number of differentiable functions of the strategy. The (marginal) fitness return through one of these functions is then defined as the sensitivity of the proxy to the strategy change in a thought experiment in which we keep the argument of all other functions unchanged. The fitness returns from state dependent decisions are usually determined from first principles conditional on the state under consideration. The epithet conditional is customarily dropped in this case. The (marginal) fitness return from a compound decision involving more than one state is calculated by summing the fitness returns for the separate states weighted with their lifetime occurrence frequencies or duration.

To keep the arguments accessible for evolutionary ecologists, we restrict our calculations from the start to the most commonly encountered class of life history models and use the simplest possible mathematical arguments rather than a more advanced functional analytical approach. In the appendices we will sketch how the same arguments can be obtained more rigorously. Basically we assume our readers to be knowledgeable only about demography and the attendant elementary results from probability theory, but not about systems theory or dynamic optimisation.

## On selection gradients, canonical equations, and evolutionarily singular strategies, a summary

Below we consider a life history model in which individuals are characterised by two dynamical variables, a physiological state, assumed to move deterministically, and a probability of still being alive, in addition to an inherited strategy *u* influencing their dynamics. The strategy *u* (or $u_\mathrm{res}$ if we talk specifically about the resident’s strategy, or $u_\mathrm{mut}$ if we talk about a mutant strategy) is supposed to be a function of the state of the individual taking values in [0, 1]. To make our life simple we assume that on the population dynamical time scale the community dynamics converges to an equilibrium, which generates the non-fluctuating environment $E_\mathrm{res}=E_\mathrm{attr}\left( u_\mathrm{res}\right)$, with $u_\mathrm{res}$ the strategy currently in residence. This assumption of a non-fluctuating environment allows us to make use of the fitness proxy $R_0\left( u_\mathrm{mut}; E_\mathrm{res}\right)$, the average lifetime offspring production of a mutant in the environment $E_\mathrm{res}$, calculated e.g. by integrating the average rate of producing offspring over the age of an individual. Consistency requires that $R_0(u_\mathrm{res};E_\mathrm{res})=1$. If $R_0\left( u_\mathrm{mut}; E_\mathrm{res}\right) >1$, a mutant has a positive probability to invade, else it cannot invade. The invasion fitness $F\left( u_\mathrm{mut}; E_\mathrm{res}\right)$ of a mutant is by definition equal to the asymptotic exponential growth rate of a mutant population in the environment $E_\mathrm{res}$ (Metz et al. 1992; Metz 2008). For $R_0$ close to 1 this invasion fitness is well approximated by

1 $$\begin{aligned} F(u_\mathrm{mut}; E_\mathrm{res})=\frac{\ln (R_0(u_\mathrm{mut};E_\mathrm{res}))}{T_\mathrm{r}\left( u_\mathrm{res}\right) }+ \mathrm{O}(\ln ^2(R_0(u_\mathrm{mut};E_\mathrm{res}))), \end{aligned}$$

where $T_\mathrm{r}\left( u_\mathrm{res}\right)$ is the average age at which the residents give birth in the environment $E_\mathrm{res}$ (Metz and Diekmann 1986; Durinx et al. 2008).

### *Remark 1*

Dieckmann et al. (2006) and Parvinen et al. (2013) consider seasonal differential-equation-based models where it is possible to calculate the invasion fitness directly by subtracting the time-averaged death rate from the time-averaged birth rate. For such models fitness takes the explicit form of an integral over the year cycle, and there is no need to fall back on an approximation. However, in the usual continuous time life history models only $R_0$ can be expressed explicitly as an integral. The availability of such an integral-based expression formed the basis for the developments in Parvinen et al. (2013), and will also be the cornerstone for our calculations.

The so-called selection gradient *G* tells how the invasion fitness of a $u_\mathrm{mut}$ close to $u_\mathrm{res}$ depends on the difference $u_\mathrm{mut}-u_\mathrm{res}$. Mathematically, the selection gradient is the derivative of the invasion fitness for $u_\mathrm{mut}$ evaluated at $u_\mathrm{mut}=u_\mathrm{res}=u$. From the previous approximation formula for the invasion fitness it follows that we can calculate *G* as

2 $$\begin{aligned} G(u)=\frac{1}{{T_\mathrm{r}}(u)} \frac{\mathrm{d}R_0}{\mathrm{d}u_\mathrm{mut}}(u; E_\mathrm{attr}(u)) \end{aligned}$$

(Durinx et al. 2008; Metz 2008). In this formula $\frac{\mathrm{d} R_0}{\mathrm{d}u_\mathrm{mut}}$ is an abstract differential quotient, i.e. a linear map transforming functions of the physiological state into a number that linearly approximates the nonlinear dependence of $R_0$ on $u_\mathrm{mut}$.

In view of our stress on life history models, let us moreover assume that *u* is an allocation, so that *u* takes values in [0, 1]. The assumptions of a non-fluctuating resident environment and a deterministically moving physiological state moreover allow us to represent the strategy *u* as a function of the age *a* of an individual, i.e., $u:{\mathbb {R}}_+\rightarrow [0,1]$. In that case we can write for a function $x:{\mathbb {R}}_+\rightarrow {\mathbb {R}}$:3(c.f., Parvinen et al. 2013). Hence the problem of calculating *G* reduces to that of calculating the function *g*. On the assumption that mutations are rare and mutational steps small the dynamics of *u* can on the evolutionary time scale be described by the so-called canonical equation (CE) of adaptive dynamics (Dieckmann and Law 1996; Champagnat 2003; Dieckmann et al. 2006; Parvinen et al. 2006, 2013; Durinx et al. 2008; Méléard and Tran 2009; Champagnat and Méléard 2011; Gupta et al. 2014)4with $T_\mathrm{s}$ the average age at which the residents die, $\sigma ^2$ the between individual variance of their offspring numbers (i.e., if $\underline{m}_i$ is a lifetime offspring number of the *i*-th individual, $\sigma ^2=\mathrm{Var}\, (\underline{m}_i)$), $\overline{n}$ their equilibrium population size, $\mu$ the (small) probability at a birth event of a mutation affecting *u*, and *c* the (small) covariance kernel of the mutational steps, i.e., if $\underline{x}$ denotes a mutational step in *u*, then

5 $$\begin{aligned} \mathrm{Cov} \left( \mathop {\int }\limits _{a_1}^{a_2}\underline{x}(\alpha )\,\mathrm{d}\alpha , \mathop {\int }\limits _{a_3}^{a_4} \underline{x}(\zeta )\, \mathrm{d}\zeta \right) =\mathop {\int }\limits _{a_1}^{a_2}\mathop {\int }\limits _{a_3}^{a_4}c(\alpha ,\zeta )\,\mathrm{d}\zeta \,\mathrm{d}\alpha . \end{aligned}$$

The form of the CE given above is the one for clonally reproducing organisms (the customary assumption in most of life history theory which, however, usually is left implicit). In Appendix 1 we briefly consider its extension to Mendelian diploids.

Our formula for the CE is slightly more complicated than the one in Dieckmann et al. (2006) and Parvinen et al. (2013). The reason is that these authors did not consider local constraints on the strategy, whereas in our case $u(a)\in [0,1]$, for each possible age $a>0$. See Appendix 2 for further information. Another difference is that Dieckmann et al. (2006) and Parvinen et al. (2013) have set the factor $\sigma ^2$ equal to 2, in keeping with the idea that for the i-models underlying the standard ordinary differential equation (ODE) models the distribution of the lifetime offspring number is geometric. Moreover, for the standard ODE models $T_\mathrm{r}=T_\mathrm{s}$ and since the *g* in Dieckmann et al. (2006) and Parvinen et al. (2013) corresponds to our $f\mathop {=}\limits ^{{\mathrm {def}}}g/T_\mathrm{r}$, the $T_\mathrm{s}$ in (4) cancels. Appendix 3 treats the corresponding considerations for the periodic ODE case considered by Dieckmann et al. (2006) and Parvinen et al. (2013), with as outcome that in this case their $\overline{n}$ should be interpreted as a harmonic death-rate weighted mean of the population sizes over a cycle.

The equilibria of the CE are the so-called ess-es. If these strategies are moreover (local) fitness maxima for the corresponding $E_\mathrm{res}$ then they are also evolutionary equilibria, to which we refer as (local) ESS-es. (An alternative is that at an attracting ess the population starts to accumulate variation, so that it no longer stays quasi-monomorphic as is supposed in the derivation of the CE. (The latter on good grounds: see Geritz et al. 2002; Geritz 2005; Dercole and Rinaldi 2008, Appendix 2).) Another way to calculate ESS-es is to maximise the invasion fitness, or alternatively $R_0$, over $u_\mathrm{mut}$, leading to a function-valued map^1^$u_\mathrm{mut}^*(u_\mathrm{res})$, followed by solving the equation $u^*_\mathrm{mut}(u_\mathrm{res})=u_\mathrm{res}$. It is here that Pontryagin’s maximum principle is encountered (e.g., Pontryagin et al. 1964; Intrilligator 1971). This principle is derived by considering the differential equations for the i-states as constraints on their time development, and to extend the idea of Lagrange multipliers as encountered in finite dimensional optimisation problems to this case. The Lagrange multipliers then become functions of time, which can be shown to satisfy a set of differential equations, and for this reason are referred to as co-states (or adjoints). In Sect. 6 we give explicit expressions for the life history models described in the next section. Appendix 5 shows how Pontryagin’s maximum principle can be derived directly from a weak variant of Bellman’s principle of optimality, which is rather better known among ecologists.

## Model ingredients

Before we get to the specifics we first introduce some notational conventions in order to keep our formulas from becoming too unwieldy.

*Conventions*

1. The argument $E_\mathrm{res}$ will be usually hidden.
2. Similarly we shall often hide the argument *u* in expressions like *P*(*a*; *u*) for the probability that an individual survives till age *a*, or *m*(*a*; *u*) for its body size at that age.
3. When we use the argument *u*, then *u* stands either for $u_\mathrm{mut}$, $u_\mathrm{res}$, or $u_\mathrm{mut}=u_\mathrm{res}$, with the context making clear which is the case.
4. For a function of a single scalar variable we use a prime to indicate its derivative. A superscript dot indicates a derivative for age, also when a function has other arguments as well.

The two dynamical variables characterising an individual are (i) one i-state variable, to wit its body mass *m*, increasing from a fixed birth mass $m(0)=m_0$, and (ii) its probability *P* to be still alive, starting from $P(0)=1$. The energy intake by an individual with body mass *m* will be denoted by *e*(*m*). The strategy of an individual will be denoted by $u:{\mathbb {R}}_+\rightarrow [0,1]:a \mapsto u(a)$, where *u*(*a*) determines which fraction of the energy intake at age *a* is used for reproduction while the remains are used for growth. The body mass just increases with $(1-u)e(m)$, while the birth rate is assumed to depend monotonically on the available energy $u\,e(m)$ as $b:{\mathbb {R}}_{+}\rightarrow {\mathbb {R}}_{+}: u(a)\,e\left( m(a)\right) \mapsto b\left( u(a)\, e\left( m(a)\right) \right)$. Finally, the energy allocation is assumed also to affect the death rate $d:[0,1] \rightarrow {\mathbb {R}}_+: u(a)\mapsto d\left( u(a)\right)$. All three functions *e*, *b*, and *d* also implicitly depend on $E_\mathrm{res}$. In this model the average lifetime offspring number of a mutant strategy $u_\mathrm{mut}$ equals

6 $$\begin{aligned} R_0&= \mathop {\int }\limits _0^{\infty } P(a)b \left( u_\mathrm{mut}(a) e\left( m(a)\right) \right) \, \mathrm{d}a, \nonumber \\ \hbox {with } m \hbox { and } P \hbox { solving }&\nonumber \\ \dot{m}&=(1-u_\mathrm{mut}) \,e(m),\quad m(0)=m_0,\nonumber \\ \dot{P}&=-d(u_\mathrm{mut}) P, \quad P(0)=1. \end{aligned}$$

Note that if $u_\mathrm{mut}=u_\mathrm{res}$, due to the value of $E_\mathrm{res}$ necessarily $R_0=1$. Moreover, we assume that the tail of *P* is bounded by a negative exponential and that *b* and *e* are bounded. These assumptions derive from the biology behind the example and imply that the improper integral in (6) exists.

## Calculating the selection gradient from a fitness-returns argument

**Fig. 1:**
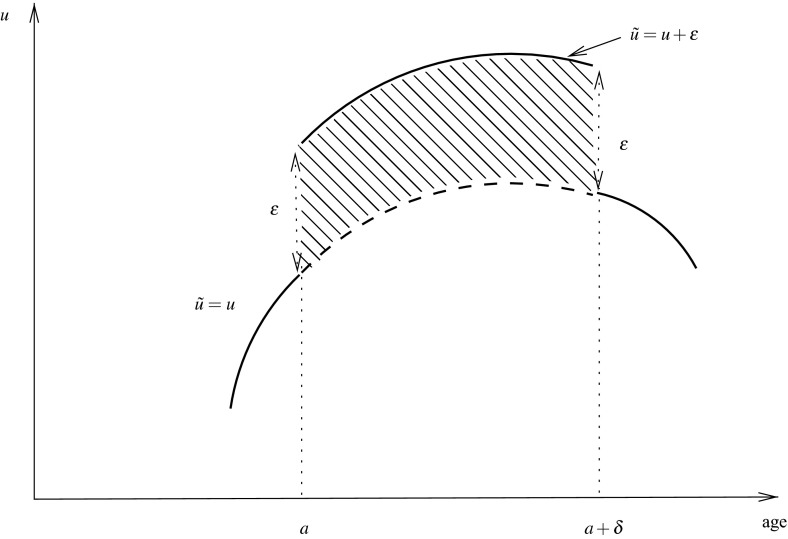
Illustration of the fitness-returns argument where *u* is any control path. The idea is to do a thought experiment in which we increase *u* between *a* and $a+\delta$ with a block *B* of height $\varepsilon$, and observe its effect on fitness

We shall express *g* from Eq. (3) in terms of the fitness returns *r*, that is, the proportional effects of small local changes in *u* on the total future reproduction. To calculate *r*(*a*; *u*) we proceed by means of a thought experiment. For a living individual aged *a* we increase *u* between *a* and $a+\delta$ by an amount $\varepsilon$, i.e., we construct a function $\tilde{u}= u+B$, $B: {\mathbb {R}}_+ \rightarrow {\mathbb {R}}$, $B(\alpha )= \varepsilon$ for $a \le \alpha < a+\delta$ and 0 elsewhere (see Fig. 1),

Calculate the resulting expected change in the expected life-time offspring number, multiply this number with $\left( \varepsilon \delta \right) ^{-1}$ and let both $\varepsilon$ and $\delta$ go to zero. Since the fitness return by definition is calculated conditional on an individual surviving to *a*, only the fraction *P*(*a*; *u*) surviving till age *a* contributes in this manner to $R_0$. Hence

7 $$\begin{aligned} g=P\, r. \end{aligned}$$

To calculate those expected additional offspring numbers we proceed in the spirit of the marginal value theorem, that is, we calculate and then add the components of *r* contributed by different routes. These components include the immediate additional number of offspring coming from the temporary increase in energy allocation to reproduction and the decreases in future offspring numbers caused by the future smaller size and lesser survival caused by the temporary decrease in allocation to growth and to staving off death. We start with the calculation of the second and third components. Let $\varDelta _{m}$ and $\varDelta _{P}$ denote the differences $m(\tilde{u})-m(u)$ and $P(\tilde{u})-P(u)$, respectively. Then, by a first-order Taylor expansion of $P(\alpha )$ and $m(\alpha )$ with respect to $u(\alpha )$, we obtain for $a\le \alpha <a+\delta$

8 $$\begin{aligned} \dot{\varDelta }_{m}&\approx (1-u)e'(m)\,\varDelta _{m}- \varepsilon \, e(m),\quad \varDelta _{m}(a)=0,\nonumber \\ \dot{\varDelta }_{P}&\approx -d(u)\, \varDelta _{P} - d'(u) \, P\, \varepsilon , \quad \varDelta _{P}(a)=0, \end{aligned}$$

which implies

9 $$\begin{aligned} \varDelta _{m}(a+\delta )&\approx -e(m(a))\varepsilon \,\delta , \quad \varDelta _{P}(a+\delta )\approx -d'(u(a))P(a)\, \varepsilon \,\delta . \end{aligned}$$

The immediate offspring gain from this strategy change over the time interval $[a,a+\delta )$ (for an individual that survived till *a*) is

10 $$\begin{aligned} \varepsilon \delta [b'(u(a)e(m(a))) e(m(a))]. \end{aligned}$$

From $a+\delta$ onwards Eq. (8) apply with $\varepsilon$ set to zero and with initial condition (9).

The future loss of offspring from this change in strategy for an individual that already has survived till *a* is

11 $$\begin{aligned} -\frac{1}{P(a)} \mathop {\int }\limits _a^{\infty } \left( \varDelta _{P}(\alpha )\,b\left( u(\alpha )\, e(m(\alpha ))\right) +P(\alpha )\,b'\left( u(\alpha )\,e(m(\alpha ))\right) u(\alpha )\, e'(m(\alpha ))\varDelta _{m}(\alpha ) \right) \mathrm{d}\alpha . \end{aligned}$$

The linearity of Eq. (8) with $\varepsilon =0$ implies that $\varDelta _\mathrm{P}(\alpha )$ and $\varDelta _\mathrm{m}(\alpha )$ are linearly dependent on the initial conditions given by (9), and therefore the outcome (11) is proportional to $\varepsilon \delta$. To make the coming calculation more transparent we introduce new functions $\hat{P}(\alpha ;a)$, $\hat{\varDelta }_m(\alpha ;a)$, $\alpha \ge a$, defined by12where $\hat{P}(\alpha ;a)$ can be interpreted as the conditional survival of an individual that has already survived to age *a*, i.e., $\hat{P}(\alpha ;a)=P(\alpha )/P(a)$, given the strategy *u*, and $\hat{\varDelta }_{m}(\alpha ;\alpha +\delta ) =\varDelta _m(\alpha )/\varDelta _m(a+\delta )$ as the relative amount by which a small perturbation in *m* present at age $a+\delta$ will propagate into the future given *u*. Similarly $\hat{P}(\alpha ;a+\delta )$ allows an alternative interpretation as relative amount by which a small perturbation in *m* present at age $a+\delta$ will propagate into the future given *u*. For $\varepsilon \downarrow 0$ and $\delta \downarrow 0$ we can then express the fitness return *r*(*a*; *u*) as follows:

13 $$\begin{aligned} r(a;u)&=b'(u(a)e(m(a)))e(m(a))\nonumber \\&\quad - e(m(a))\mathop {\int }\limits _a^{\infty }{\hat{P}}(\alpha ;a) b'(u(\alpha )e(m(\alpha ))) u(\alpha )e'(m(\alpha ))\hat{\varDelta }_{m}(\alpha ;a)\mathrm{d}\alpha \nonumber \\&\quad -d'(u(a))\mathop {\int }\limits _a^{\infty } {\hat{P}}(\alpha ;a)\,b(u(\alpha )e(m(\alpha )))\mathrm{d}\alpha . \end{aligned}$$

At an ess $u^*$ the return *r*(*a*) should be 0 when $u^*(a)\in (0,1)$, non-positive when $u^*(a)=0$ and non-negative when $u^*(a)=1$.

## The other ingredients of the canonical equation

To complete the canonical equation we need to find expressions for $T_\mathrm{r}$, $T_\mathrm{s}$, and $\sigma ^2$. Since $R_0(u;E_\mathrm{attr}(u))=1$, the expression $P(\alpha ;u) b(u(\alpha ) e(m(\alpha ;u)))$ is a probability density. Furthermore,

14 $$\begin{aligned} -\mathop {\int }\limits _0^\infty \dot{P}(\alpha ;u)\, \mathrm{d} \alpha = P(0)-\mathop {\lim }\limits _{A \rightarrow \infty }P(A)=1, \end{aligned}$$

thus also $-\dot{P}(\alpha ;u)$ is a probability density. Therefore, $T_\mathrm{r}$ and $T_\mathrm{s}$ can be expressed directly:

15 $$\begin{aligned} T_\mathrm{r}(u)&=\mathop {\int }\limits _0^{\infty } \alpha P(\alpha ;u) b(u(\alpha ) e(m(\alpha ;u))) \mathrm{d}\alpha , \end{aligned}$$

16 $$\begin{aligned} T_\mathrm{s}(u)&=-\mathop {\int }\limits _0^{\infty } \alpha \dot{P}(\alpha ;u)\mathrm{d}\alpha =-\lim _{A\rightarrow \infty }\left. \alpha P(\alpha )\right| _{\alpha =0}^{A}+\mathop {\int }\limits _0^{\infty } P(\alpha ;u)\mathrm{d}\alpha = \mathop {\int }\limits _0^{\infty } P(\alpha ;u)\mathrm{d}\alpha , \end{aligned}$$

where the last equation comes from integrating by parts and using $\mathop {\lim }\nolimits _{A\rightarrow \infty }\left( A P(A)\right) =0$.

To calculate $\sigma ^2$ we have to be more specific about the microstructure of the reproduction process. The assumption that naturally leads to (5) is that for an individual that is still alive the births come in a Poisson process with rate *b*(*ue*(*m*)), or, slightly more generally, in clutches of average size *C*(*u*, *e*(*m*)) produced according to a Poisson process with rate $\frac{b(ue(m))}{C(u,e(m))}$. We confine ourselves here to the former option. In such a case, for a given age at death *a* the total offspring number is Poisson distributed with mean

17 $$\begin{aligned} \lambda (a;u)=\mathop {\int }\limits _0^a b\left( u(\alpha )e(m(\alpha ;u))\right) \mathrm{d}\alpha . \end{aligned}$$

In general, *a* is a realisation of a random variable $\underline{a}$. Hence, the lifetime offspring number is a mixture of Poisson random variables. The mean of $\underline{\lambda }=\lambda (\underline{a};u)$ is nothing but the average lifetime offspring number^2^

18 $$\begin{aligned} {\mathbf {E}}(\underline{\lambda }) = R_0 \left( u; E_\mathrm{attr}(u)\right) =1. \end{aligned}$$

Therefore, $$\sigma _{\lambda }^2 ={\mathbf {E}}(\lambda ^2)-({\mathbf {E}}(\lambda ))^2={\mathbf {E}}(\lambda ^2)-1$$, with

19 $$\begin{aligned} {\mathbf {E}}(\underline{\lambda }^2) = -\mathop {\int }\limits _0^{\infty }\dot{P} (\alpha ;u) \,\lambda (\alpha ;u) \mathrm{d}\alpha = 2\mathop {\int }\limits _0^{\infty }\lambda (\alpha ;u)P(\alpha ;u) b(u(\alpha ) e(m(\alpha ;u))) \mathrm{d}\alpha \end{aligned}$$

(integration by parts).

Finally, from the general rules for mixtures of distributions^3^

20 $$\begin{aligned} \sigma ^2=\sigma _{\lambda }^2+1={\mathbf {E}}(\underline{\lambda }^2). \end{aligned}$$

## Locating fitness maxima by means of Pontryagin’s maximum principle

The equilibria of the canonical equation are called ess-es. The reason for this from a differential equations viewpoint unusual terminology is that among the ess-es only the ESSes, characterised by the fact that they are also maxima of the current fitness landscapes, are immune to evolutionary change. One way of calculating ESSes for life history problems is to make use of Pontryagin’s maximum principle to locate the fitness maxima in $u_\mathrm{mut}$ that go with a given $u_\mathrm{res}$ and then to set $u_\mathrm{mut}=u_\mathrm{res}$.

In contrast to the canonical equation, Pontryagin’s maximum principle, at least in its original form and with a number of standard assumptions satisfied, is textbook material. In this section we will just in the wake of Intrilligator (1971) state the conditions that an optimal *u* has to satisfy. For a discussion of different variants of Pontryagin’s maximum principle and its connection to the Bellman’s principle of optimality (Bellman 1957), see Appendix 5. In the notation of Intrilligator (1971), Eq. (6) can be rewritten in the following form:

21 $$\begin{aligned} {\mathbf {x}}=\left( \begin{array}{c} m\\ P \end{array}\right) , \quad I \left( {\mathbf {x}},u\right) =P\, b(u e(m)), \quad J=R_0, \quad {\mathbf {f}}({\mathbf {x}},u)=\left( \begin{array}{c} (1-u)e(m)\\ -d(u)P \end{array}\right) , \end{aligned}$$

with ${\mathbf {x}}$ the state vector, *J* the quantity to be optimised, calculated as the lifetime integral of *I*, and ${\mathbf {f}}$ the right hand side of the differential equation for ${\mathbf {x}}$. Pontryagin’s maximum principle then says that if $u^*$ maximises *J*, then at each age $a\in [0,\infty )$ it also maximises the so-called Hamiltonian, defined as

22 $$\begin{aligned} H\mathop {=}\limits ^{{\mathrm {def}}}I + {\mathbf {y}}^T {\mathbf {f}}, \end{aligned}$$

with $${\mathbf {y}}=\left( \begin{array}{c}y_1 \\ y_2\end{array}\right) $$ being the so-called co-state (or adjoint) vector, where its components satisfy the differential equations

23 $$\begin{aligned} \dot{y}_1=-\frac{\partial \,H}{\partial \,m}, \quad \dot{y}_2=-\frac{\partial \,H}{\partial \,P}, \end{aligned}$$

with final conditions $\mathop {\lim }\nolimits _{A\rightarrow \infty } y_1(A)=\mathop {\lim }\nolimits _{A\rightarrow \infty } y_2(A)=0$. If $u^*$ maximises $R_0$ in (6), then it also maximises

24 $$\begin{aligned}&H(m(a),P(a),u(a))= P(a)\,b\left( u(a) e\left( m(a)\right) \right) \nonumber \\&\quad +y_1(a) \left( 1-u(a)\right) e\left( m(a)\right) - P(a)\,y_2(a) d\left( u(a)\right) ,\nonumber \\&\hbox {subject to}&\nonumber \\&\dot{y}_1 = - \left( 1-u \right) e'\left( m \right) y_1 - P b'\left( u e\left( m \right) \right) u e'\left( m \right) , \nonumber \\&\quad \dot{y}_2 = d\left( u\right) y_2 - b\left( u e\left( m \right) \right) ,\nonumber \\&\dot{m}=\left( 1-u \right) e\left( m \right) , \quad \dot{P}=-d\left( u \right) P , \nonumber \\&\mathop {\lim }\limits _{A\rightarrow \infty } y_1(A)=\mathop {\lim }\limits _{A\rightarrow \infty } y_2(A)=0, \quad m(0)=m_0, \quad P(0)=1. \end{aligned}$$

In other words, if *J* is maximised by $u^*$, then

25 $$\begin{aligned} u^*(a)=\mathrm{arg}\mathop {\max }\limits _{u(a)\in [0,1]} H\left( m(a),P(a),u(a)\right) , \end{aligned}$$

at each $a\in [0,\infty )$. This implies that

26 $$\begin{aligned}&g_H(a)\mathop {=}\limits ^{{\mathrm {def}}}\frac{\partial H(m(a),P(a),u(a))}{\partial u(a)} =P(a) b'\left( u(a)\, e(m(a))\right) e\left( m(a)\right) \nonumber \\&\quad -y_1(a) e\left( m(a)\right) - y_2(a)d'\left( u(a)\right) P(a)=0 \nonumber \\&\quad \quad \;\; {\mathrm {when}}\;\; u^*(a)\in (0,1), \nonumber \\&g_h(a)\ge 0 \quad {\mathrm {when}} \;\; u^*(a)=1, \nonumber \\&g_h(a)\le 0 \quad {\mathrm {when}} \;\; u^*(a)=0. \end{aligned}$$

Obviously, to assure that $u^*(a)$ is a local maximum of *J*, resp. *H*, the derivative of $g_H(a)$ with respect to *u*(*a*) has to be negative whenever $0<u(a)<1$.

The co-states $y_1(a)$ and $y_2(a)$ in (26) can be expressed from (24) as follows:

27 $$\begin{aligned} \nonumber y_1(a)&=y_1(0) {\exp }\left( -\mathop {\int }\limits _0^a (1-u(\alpha )) e'(m(\alpha )) \mathrm{d}\alpha \right) \nonumber \\&\quad -\mathop {\int }\limits _0^a (P(\alpha ) \, b'(u(\alpha ) \,e(m(\alpha )))u(\alpha ) e'(m(\alpha ))) \, {\exp }\left( -\mathop {\int }\limits _{\alpha }^a(1-u(\tau ))e'(m(\tau ))\mathrm{d}\tau \right) \mathrm{d} \,\alpha \nonumber \\ y_2(a)&=y_2(0) {\exp }\left( -\mathop {\int }\limits _0^a d(u(\alpha )) \mathrm{d}\alpha \right) \nonumber \\&\quad -\mathop {\int }\limits _0^a b\left( u(\alpha ) \,e\left( m(\alpha )\right) \right) \, {\exp }\left( \mathop {\int }\limits _{\alpha }^a(d(u(\tau )))\mathrm{d}\tau \right) \mathrm{d} \,\alpha , \end{aligned}$$

where $y_1(0)$ and $y_2(0)$ have to be chosen such that $\mathop {\lim }\nolimits _{A\rightarrow \infty } y_1(A)=\mathop {\lim }\nolimits _{A\rightarrow \infty } y_2(A)=0$. In Appendix 4 we show that:

(i) 28 $$\begin{aligned} \frac{y_1(a)}{P(a)}=\mathop {\int }\limits _a^{\infty } (\hat{P}(\alpha ;a) \, b'(u(\alpha ) \,e\left( m(\alpha )\right) )u(\alpha ) e'(m(\alpha ))) \varDelta _m (\alpha )\, \mathrm{d}\alpha , \end{aligned}$$
  which can be interpreted as the marginal loss or gain per unit weight change (sensitivity) of lifetime offspring due to lower subsequent weights, and
(ii) 29 $$\begin{aligned} \frac{y_2(a)}{P(a)}=\mathop {\int }\limits _a^{\infty } (\hat{P}(\alpha ;a) \, b(u(\alpha ) \,e\left( m(\alpha )\right) ) \, \mathrm{d}\alpha , \end{aligned}$$
  which can be interpreted as the sensitivity of lifetime offspring due to lower subsequent survival. Moreover, in the same appendix we show that Formulas (12)–(13) for calculating the fitness returns (c.q. the selection gradient) and Formulas (26)–(27) for the derivative of the Hamiltonian with respect to *u*, are equivalent.

The detailed match between the results from the two approaches more generally follows from the correspondence between Bellman’s principle of optimality and Pontryagin’s maximum principle that we work out in some detail in Appendix 5.

On the practical side we point at the fact that even when one is only interested in calculating an ESS with the help of Pontryagin’s maximum principle, and has no particular interest in the evolutionary trajectories by which this ESS may be reached, running some discretised variant of the canonical equation can still provide an effective computational implementation of that principle as used in ESS calculations.

## Discussion

The main contribution of this note is that we carefully set up the CE for life history decisions. As it turned out, a few details had to be added to the exposition in Parvinen et al. (2013). In particular, it was necessary to extend the canonical equation so as to be able to handle inequality constraints. In addition, there was the small detail of the appearance of an additional multiplicative factor accounting for the difference in the initial branching process that mutants have to get through before getting established compared to the linear birth and death process that appears in this role for ODE population models (c.f. Durinx et al. 2008).

Given the venerable history of Pontryagin’s maximum principle and its applications to life history theory it should raise no wonder that interpreting the co-states is not new. In particular, Jesus Alberto Leon already did so in the nineteen-seventies (Leon 1976); see also Perrin and Sibly (1993). However, in those days there was no canonical equation around and hence no need to make a connection. Moreover, these early authors put forward the interpretation seemingly ex cathedra, and only post hoc and summarily related it to a marginal value argument, without exhibiting the explicit connection made in our Sects. 4 and 6 and Appendices 4 and 5. In particular, they did not consider “co-state variables” for other *u* than the optimal one. Precisely these “generalised co-state variables” occur as ingredients of the selection gradient. Although such variables are already used in numerical approaches to Pontryagin’s maximum principle (e.g. Näslund et al. 1974), we believe that our explicit calculations add to the biological understanding of the mathematical structure of eco-evolutionary models.

As a final point we note that the argument that we provide in Sect. 5, although this was not spelled out there, is exemplary of a more general principle. When we delve a little more deeply into the stochastic models for individual behavior, as was necessary in order to calculate $\sigma ^2$, it generally becomes clear how embarrassingly oversimplified such models tend to be. In our case it turned out that it was implicitly assumed that microscopically the production of young is coupled far more loosely to the energy flow to reproduction than seemingly is assumed at the macroscopic level. Real organisms first have to accumulate the necessary energy that then is transformed into the birth of a young, instead of randomly producing young on the basis of the instantaneous availability of resources. Therefore in reality the production of young usually is far more regular than Poisson (so that $\sigma ^2$ is close to $\sigma _{\lambda }^2$ ), and at a given time depends also on past energy availabilities. Hence the idea that the average rate of offspring production at age *a* is just a function *b* of $u(a)\, e (m(a) )$ is at best only a rough approximation. One possible justification is that most of the time $u\, e(m)$ varies only slowly compared to the rate at which young are produced, and that if reproduction does occur spread out in time, no two individuals will be in the same phase of their reproduction cycle, so that at any one time the effective offspring production of the individuals that have a size close to the scalar $\overline{m}$ may well be on average close to $b(ue(\overline{m}))$. However, the modelling community is still a long way from proving any rigorous approximation theorems of this ilk. (See Heijmans and Metz (1989) for another possible justification, which, however, is less often applicable in a general life history context.) Of course we also made other simplifying assumptions, like neglecting basal metabolism. However, these simplifications were only put in to ease the exposition, raise no deep mathematical issue, and hence can presumably be relaxed without great difficulty.

## Figures and Tables

**Fig. 2:**
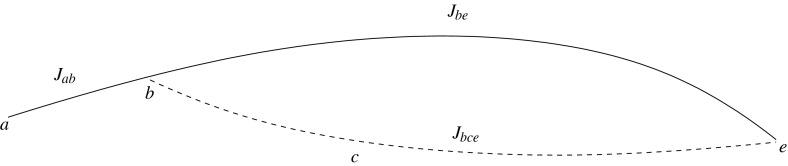
Illustration of Bellman’s optimality principle

**Fig. 3:**
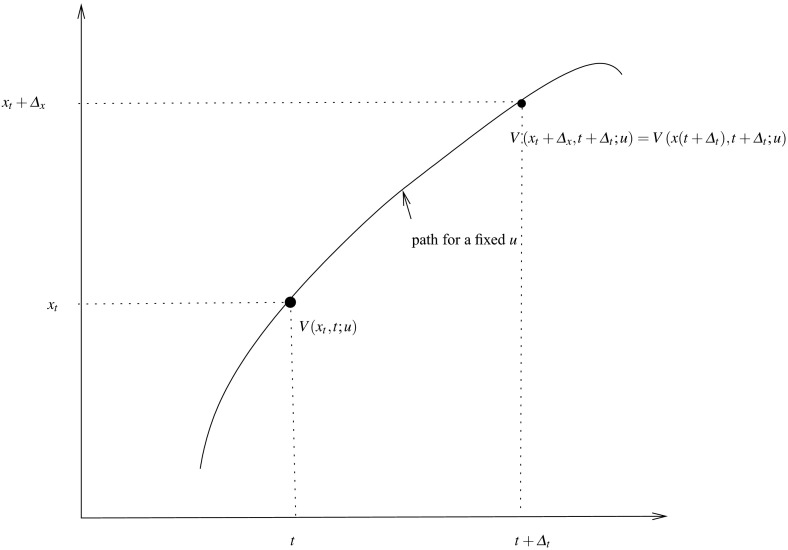
A path for a specific choice of *u* in the time-state space

## Appendix 1: Mendelian organisms

Most life history models implicitly assume clonal reproduction. Yet, by far the majority of organisms that are supposedly targeted by these modeling efforts are Mendelian diploids (c.f. Stearns 1976, 1977). To help overcoming this awkward discrepancy we summarise here some results for the Mendelian case (c.f. Metz and de Kovel 2013).

The first difference between the clonal and Mendelian cases is that the homozygote phenotype present after a substitution differs from the heterozygote phenotype that invaded. Since for small mutational steps the genotype to phenotype map is approximately additive, this leads to the appearance of an additional factor two (on the assumption that there are no parental effects) in the right hand side of the canonical equation.

A more fundamental difference is that as a rule the gametes involved in sexual reproduction come in two types, macro- and micro-gametes. To keep the discussion simple we concentrate on the case where the sexes are separate, for otherwise we have to consider triple allocation targets, to growth, macro-gametes, and micro-gametes. In the case of separate sexes we simply have $u=(u_\mathrm{f},u_\mathrm{m})$, with $u_\mathrm{f}$ the allocation rule of the females, and $u_\mathrm{m}$ the one of the males. These allocation rules in general will be evolutionarily coupled through mutational co-variances, but, except for a common time scaling with $T_\mathrm{r}^{-1}(u)$, the selection gradients can be treated separately, as if we were dealing with two coevolving species, with each of the sexes setting part of the environment, which now also includes fertilization opportunities, for the other sex. This independence derives from the additive relation $R_0=\frac{1}{2} (R_\mathrm{f}+R_\mathrm{m})$ , with $R_\mathrm{f}$ the average lifetime number of kids of a female and $R_\mathrm{m}$ the average lifetime number of kids of a male (e.g. Metz and Leimar 2011; Gyllenberg et al. 2011). Similarly, $T_\mathrm{r}=\frac{1}{2}(T_\mathrm{r,f}+T_\mathrm{r,m})$, where the additional indices $\mathrm{f}$ and $\mathrm{m}$ mean that the so indexed quantity, in this case the average age of the parent at the birth of its kids, is calculated conditional on the sampled individual being a female or a male. Hence, for $\mathrm{S}\in \{\mathrm{f},\mathrm{m}\}$,

30 $$\begin{aligned} G_\mathrm{S}(u)=\frac{\partial \,F}{\partial \, u_{\mathrm{S}, \mathrm{mut}}}\left( u;E_\mathrm{attr}(u)\right) =\frac{1}{T_{\mathrm{r},\mathrm{f}}+T_{\mathrm{r},\mathrm{m}}} \,\frac{\mathrm{d}R_\mathrm{S}}{\mathrm{d} u_{\mathrm{S},\mathrm{mut}}} \left( u; E_\mathrm{attr}(u)\right) . \end{aligned}$$

The action of the derivative can again be expressed as an integral

31 $$\begin{aligned} \frac{\mathrm{d} R_\mathrm{S} (u; E_\mathrm{attr}(u))}{ \mathrm{d} u_{\mathrm{S},\mathrm{mut}}}x=\mathop {\int }\limits _0^{\infty } g_\mathrm{S} (\alpha ;u) \, x(\alpha ) \, \mathrm{d}\alpha , \end{aligned}$$

with the functions $g_\mathrm{S}$ calculated in the same manner as for the clonal model, with the hidden argument $E_\mathrm{attr}$ in the functions $b_\mathrm{S}$ accounting for any differences in availability of fertilization opportunities at different $u_\mathrm{res}$. Finally, $T_\mathrm{s}=q_\mathrm{f}\, T_{\mathrm{s},\mathrm{f}} + q_\mathrm{m}\, T_{\mathrm{s},\mathrm{m}}$, with $q_\mathrm{f}$ and $q_\mathrm{m}$ the relative frequencies with which the sexes are born into the resident population, and $\sigma ^2=\frac{1}{4}(q_\mathrm{f}\, \sigma ^2_\mathrm{f}+ q_\mathrm{m} \sigma ^2_\mathrm{m}+q_\mathrm{f}^{-1}+q_\mathrm{m}^{-1}-2)$ (the latter formula also takes into account the random sampling of alleles during the offspring production by the heterozygotes).

The upshot is that the males and females in any ESS-es satisfy separate Pontryagin maximum principles, with the coupling between the sexes appearing in the equations only through the influences the resident female and male strategies exert on $E_\mathrm{attr}$. Yet, the fact that the fertilization opportunities come as a component of $E_\mathrm{attr}$ inextricably entwines life history evolution with sex ratio evolution.

## Appendix 2: How to deal with local constraints?

This appendix emulates the treatment in Dieckmann et al. (2006) with the difference that we go one step further in working out the result, so that we end up with a simple formula that provides the right match for the Pontryagin maximum principle.

For a start we note that in principle the mutational covariance function is not constant over evolutionary time, but depends on the evolutionary history of the population. In particular, the distribution of mutational steps has to change near a constraint boundary so as to preclude overstepping it. There are various ways in which this change may happen. Most of these will make the distribution of the steps asymmetric, with close to the boundary steps towards the interior of the space of feasible strategies becoming more common relative to steps towards the boundary. The CE as given by Dieckmann et al. (2006) and Parvinen et al. (2013) is based on the assumption that the mutation distribution is symmetric, in line with most papers on the CE; (Formulas for the non-symmetric case may be found in Dieckmann and Law (1996), Champagnat (2003), and Champagnat and Méléard (2011).) In our formula we have kept the form of the CE unchanged in the interior of the constraint set and only set the right hand side equal to zero where that movement would lead to the passing of a constraint boundary. The rationale for this ploy is the following. The CE is derived as a limit in which one lets a factor that scales the mutational steps go to zero. This means that at any distance from the constraint boundary eventually the effect of the constraint will no longer be felt, and if the mutation distribution would otherwise be symmetric, this symmetry would eventually be recovered for all resident strategies that are not located on the boundary. At boundary strategies, in the CE limit the movement component in the outward direction has to drop to zero, since there the mutation distribution stays forever asymmetric, with its probability mass all located on the feasible side. In the limit the distribution of this mass contracts towards the boundary. On segments of the boundary where the nearby movement is towards that boundary the movement on the boundary becomes restricted to it by the covariance function abruptly becoming singular. On the natural assumption that the constraint does not affect movement parallel to the boundary, this corresponds to just setting the right hand side to zero at the indicated values of *a*. (In finite dimensional trait spaces the analogous condition is that on the boundary the movement component orthogonal to the boundary becomes zero whenever close by the movement is towards the boundary, while the movement component parallel to the boundary is a continuous extension of the movement component in that direction in the interior of the constraint set.)

## Appendix 3: The canonical equation for periodic ODE population models

The right hand side of the CE equals

32 $$\begin{aligned}&[\hbox {Rate at which mutants are produced}] \times \hbox { average of } [ \hbox { the effect of a mutation times a } \nonumber \\&\hbox {linear approximation for the probability that mutant invades}]. \end{aligned}$$

On the assumption of small mutational steps and a symmetric mutation distribution the latter average gives $\frac{1}{2}$ times the mutational covariance operator applied to the selection gradient, where the $\frac{1}{2}$ comes from the fact that the linear approximation only applies in the half space where the invasion fitness is positive and is replaced by 0 where it is negative. The factor $\sigma ^{-2}$ in (4) comes from the lowest order term of the asymptotic expansion for the probability *Q* that a mutant with a slightly positive fitness $(0{<}F{\ll } 1)$ invades. When births occur singly the individual-based models underlying ODE population models can for the initial phases of mutant invasion be approximated by a linear birth and death process. For constant environments the corresponding generation process is of Galton–Watson type with a geometric offspring distribution with mean $R_0=\frac{b}{d}$ with *b* and *d* the per capita birth and death rates of the mutant, respectively, while $F=b-d=\frac{R_0-1}{T_\mathrm{s}}$. Hence the invasion probability equals $Q=1-R_0^{-1}=R_0-1+{\mathrm {O}}((R_0-1)^2)$. More in general, $Q=2\,\sigma _\mathrm{e}^{-2} \, \ln \, R_0 + {\mathrm {O}}(\ln ^2 \,R_0)$, with $\sigma _\mathrm{e}^2$ a measure for the average variability of the offspring production of the residents (for which $R_0=1$), which in the case of a single birth state reduces to the variance of the offspring distribution $\sigma ^2$ (c.f. Metz and de Kovel 2013; Durinx et al. 2008). (For a geometric distribution with mean 1: $\sigma ^2=2$.) The rate at which mutants are produced equals the population birth rate times the per birth probability of a mutation. The factor $\overline{n}$ in (4) appears by re-expressing the population birth rate *B* of the resident as $\frac{\overline{n}}{T_\mathrm{s}}$, based on the general consistency relation $\overline{n}=B\, T_\mathrm{s}$. Below we consider the extension of these considerations to periodic environments; the further extension to general ergodic environments is treated in Ripa and Dieckmann (2013).

In the case of periodic environments we have to average both the number of births as well as the probability to invade over a cycle, where the first average is a time average and the latter average is over the distribution of births over the cycle.

To calculate the invasion probability in dependence of the phase $\theta$ of appearance of a mutant during the environmental cycle, $q(\theta )$, we use the general formula for the invasion probability for linear birth and death processes with time variable parameters derived in Kendall (1948):

33

With time rescaled so that the period equals $T=1$, we then get

34 $$\begin{aligned} Q=\mathop {\int }\limits _0^1 q(\theta )\, w(\theta )\, \mathrm{d}\theta , \end{aligned}$$

with

35 $$\begin{aligned} w(\theta )=\frac{b_0(\theta )\mathrm{e}^{r_0(\theta ;0)}}{\mathop {\int }\nolimits _0^1 b_0(\tau )\,\mathrm{e}^{r_0(\theta ;0)}\mathrm{d}\tau } \end{aligned}$$

the probability distribution of the phase of the environmental cycle at which a mutant may be expected to appear, with $b_0$ and $d_0$ the periodic per capita birth and death rates of the residents and $r_0$ defined as in (33).

The stationarity of the resident population implies that $r_0(t+1;t)=0$, i.e., $\mathop {\int }\nolimits _t^{t+1} b_0 (\tau )\, \mathrm{d}\tau = \mathop {\int }\nolimits _t^{t+1} d_0 (\tau ) \, \mathrm{d}\tau$ (no per capita population growth over a full environmental cycle) as well as $\mathop {\int }\nolimits _{t}^{t+1} b_0(\tau ) \, \mathrm{e}^{r_0(\tau ;t)} \mathrm{d}\tau =\mathop {\int }\nolimits _t^{t+1} d_0(\tau ) \, \mathrm{e}^{r_0(\tau ;t)} \, \mathrm{d}\tau$ (the total births over a cycle matches the death toll over the cycle). More in general $F=r(t+1;t)=r(1;0)$ and

36 $$\begin{aligned} R_0=\frac{\overline{b}}{\overline{d}}, \end{aligned}$$

with

37 $$\begin{aligned} \overline{b}\mathop {=}\limits ^{{\mathrm {def}}}\mathop {\int }\limits _0^1 b(\tau )\, \mathrm{d}\tau = \mathop {\int }\limits _t^{t+1} b(\tau )\, \mathrm{d}\tau \quad \hbox {and} \quad \overline{d}\mathop {=}\limits ^{{\mathrm {def}}}\mathop {\int }\limits _0^1 d(\tau ) \mathrm{d}\tau = \mathop {\int }\limits _{t}^{t+1} d(\tau ) \, \mathrm{d} \tau \end{aligned}$$

(Bacaer and Guernaoui 2006), where in the periodic case $R_0$ is defined as the dominant eigenvalue of the operator that gives the average number of newborns born at different phases of the cycle for mothers born at different phases. To calculate the derivative of *Q* we introduce a scalar variable *x* by which we parametrise a curve in the space of strategies passing transversally through the resident value at $x=0$, and write all the coefficient functions as functions of *x*, written as an index in the case of *b*, *d*, and *r*. As later on we also need the invasion probability and invasion fitness as a function of any mutant strategy, we will denote the maps from *x* to these two quantities as $\tilde{Q}$ and $\tilde{F}$. With

38 $$\begin{aligned} M(x)\mathop {=}\limits ^{{\mathrm {def}}}\mathop {\int }\limits _0^{\infty } \mathrm{e}^{-r_\mathrm{x}(\alpha ;0)} \, d_\mathrm{x}(\alpha ) \, \mathrm{d}\alpha \end{aligned}$$

we can write

39 $$\begin{aligned} q(\theta ;x) =\frac{1}{1+\mathop {\int }\nolimits _{\theta }^1 \mathrm{e}^{-r_\mathrm{x}(\alpha ;\theta )} \, d_\mathrm{x}(\alpha )\,\mathrm{d}\alpha +\mathrm{e}^{-r_\mathrm{x}(1;\theta )}\, M(x)}. \end{aligned}$$

From $\mathop {\lim }\nolimits _{x\rightarrow 0} q(\theta ;x)=0$ it follows that $\mathop {\lim }\limits _{x\rightarrow 0} M(x)=\infty$. Hence

40 $$\begin{aligned} \frac{\partial \,q}{\partial \,x}(\theta ;0)=-\mathrm{e}^{r_0(1;\theta )} \mathop {\lim }\limits _{x\rightarrow 0} \frac{M'(x)}{M^2(x)} \end{aligned}$$

and

41 $$\begin{aligned} \tilde{Q}'(0)=-\frac{\mathop {\int }\nolimits _0^1 b_0(\theta )\, \mathrm{d}\theta }{\mathop {\int }\nolimits _0^1 b_0(\theta )\,\mathrm{e}^{r_0(\theta ;0)}\, \mathrm{d}\theta }\, \mathop {\lim }\limits _{x\rightarrow 0 }\frac{M'(x)}{M^2(x)}. \end{aligned}$$

To calculate the term after the limit sign we observe that

42 $$\begin{aligned} M(x)\mathop {=}\limits ^{{\mathrm {def}}}\mathop {\int }\limits _0^1 \mathrm{e}^{-r_\mathrm{x}(\theta ;0)} d_\mathrm{x} (\theta ) \, \mathrm{d}\theta + \mathrm{e}^{-r_\mathrm{x}(1;0)} M(x). \end{aligned}$$

Hence

43 $$\begin{aligned}&M(x)=\frac{\mathop {\int }\nolimits _0^1 \mathrm{e}^{-r_\mathrm{x}(\theta ;0)} \,d_\mathrm{x}(\theta )\, \mathrm{d}\theta }{1-\mathrm{e}^{-r_\mathrm{x}(1;0)}}, \end{aligned}$$

44 $$\begin{aligned}&\mathop {\lim }\nolimits _{x\rightarrow 0} \frac{M'(x)}{M^2(x)}=\frac{-\tilde{F}'(0)}{ \mathop {\int }\limits _0^1 \mathrm{e}^{-r_0(\theta ;0)} \, d_0(\theta )\, \mathrm{d}\theta } \end{aligned}$$

and

45 $$\begin{aligned} \tilde{Q}'(0)=\frac{\mathop {\int }\nolimits _0^1 b_0(\theta )\, \mathrm{d} \theta }{ \mathop {\int }\nolimits _0^1 b_0(\theta )\,\mathrm{e}^{r_0(\theta ;0)}\, \mathrm{d} \theta \mathop {\int }\nolimits _0^1 \mathrm{e}^{-r_0(\theta ;0)} d_0(\theta )\,\mathrm{d}\theta } \tilde{F}'(0). \end{aligned}$$

Hence away from local constraints the CE becomes

46 $$\begin{aligned} \frac{\mathrm{d}s}{\mathrm{d}t}&=\frac{1}{2} \mu \mathop {\int }\nolimits _0^1 b_0(\alpha ) \tilde{n} (\alpha ) \, \mathrm{d}\alpha \,\frac{\mathop {\int }\nolimits _0^1 b_0(\alpha )\,\mathrm{d}\alpha }{\mathop {\int }\nolimits _0^1 b_0(\alpha ) \, \mathrm{e}^{r_0(\alpha ;0)}\, \mathrm{d} \alpha \, \mathop {\int }\nolimits _0^1 \mathrm{e}^{-r_0(\alpha ;0)}\, d_0(\alpha )\,\mathrm{d}\alpha }\nonumber \\&\quad \times \mathop {\int }\nolimits _0^1 c(\theta ,\alpha ) \, f(\alpha ;s)\, \mathrm{d}\alpha \nonumber \\&=\frac{1}{2}\mu \frac{\mathop {\int }\nolimits _0^1 d_0(\alpha )\,\mathrm{d}\alpha }{\mathop {\int }\nolimits _0^1 \tilde{n}^{-1}(\alpha ) d_0(\alpha ) \mathrm{d}\alpha } \mathop {\int }\nolimits _0^1 c(\theta ,\alpha )\, f(\alpha ;s)\,\mathrm{d}\alpha \end{aligned}$$

where *s* now denotes the strategy, which in the seasonal flowering model of Dieckmann et al. (2006) consists of a flowering intensity as a function of $\theta$, and

47 $$\begin{aligned} f(\theta ;s) = \frac{\mathrm{d}\left( b(\theta )-d(\theta )\right) }{\mathrm{d} \, s_\mathrm{mut} (\theta )}\left( s;E_\mathrm{attr}(s)\right) . \end{aligned}$$

Hence the $\overline{n}$ in Parvinen et al. (2013) has to be interpreted as

48 $$\begin{aligned} \frac{\mathop {\int }\nolimits _0^1 d_0 (\alpha )\, \mathrm{d}\alpha }{\mathop {\int }\nolimits _0^1 \tilde{n}^{-1} (\alpha )\, d_0(\alpha )\,\mathrm{d}\alpha }. \end{aligned}$$

To see how (46) compares with (4) we first observe that for periodically fluctuating populations there is no immediate counterpart for the equality $\overline{n}=B\,T_\mathrm{s}$, so we substitute the latter in (4), while observing that the counterpart of *B* in (46) is $\mathop {\int }\nolimits _0^1 b_0(\alpha )\tilde{n}(\alpha )\, \mathrm{d}\alpha$. After substituting $f(\alpha ;s)=\frac{g(\alpha ;s)}{T_\mathrm{r}(s)}$ in (46) we then end up with the pairing

49 $$\begin{aligned} \frac{2\,T_\mathrm{r}}{\sigma _e^2}=\frac{\mathop {\int }\nolimits _0^1 b_0(\alpha )\, \mathrm{d}\alpha }{\mathop {\int }\nolimits _0^1 b_0(\alpha )\, \mathrm{e}^{r_0(\alpha ;0)}\,\mathrm{d}\alpha \, \mathop {\int }\nolimits _0^1 \mathrm{e}^{-r_0(\alpha ;0)} \, d_0(\alpha )\,\mathrm{d}\alpha }. \end{aligned}$$

To calculate $T_\mathrm{r}$ we use $F=\overline{b}-\overline{d}$ and $R_0=\overline{b}/\overline{d}$ together with (1) to find

50 $$\begin{aligned} T_\mathrm{r}&=\overline{d}_0^{-1}=\overline{b}_0^{-1}. \end{aligned}$$

Therefore,

51 $$\begin{aligned} \sigma _e^2&=\frac{2\mathop {\int }\nolimits _0^1 b_0(\alpha )\, \mathrm{e}^{r_0(\alpha ;0)} \,\mathrm{d}\alpha }{\mathop {\int }\nolimits _0^1 b_0(\alpha )\, \mathrm{d}\alpha }\cdot \frac{\mathop {\int }\nolimits _0^1 \mathrm{e}^{-r_0(\alpha ;0)}d_0(\alpha )\, \mathrm{d}\alpha }{ \mathop {\int }\nolimits _0^1 d_0(\alpha )\, \mathrm{d}\alpha } \nonumber \\&= \frac{2\, \mathop {\int }\nolimits _0^1 \mathrm{e}^{r_0(\alpha ;0)} \, d_0(\alpha )\, \mathrm{d}\alpha \mathop {\int }\nolimits _0^1 \mathrm{e}^{-r_0(\alpha ;0)}\, d_0(\alpha )\, \mathrm{d}\alpha }{\left( \mathop {\int }\nolimits _0^1 d_0(\alpha )\, \mathrm{d}\alpha \right) ^2}. \end{aligned}$$

## Appendix 4: Relating the results of Sects. 4 and 6

In this appendix we show that formulas (12)–(13) for calculating the fitness returns (c.q. the selection gradient) and formulas (27)–(26) for the derivative of the Hamiltonian with respect to *u* are equivalent. To enhance the similarity divide $g_H$ by *P* and set $\tilde{y}_1(a)\mathop {=}\limits ^{{\mathrm {def}}}P^{-1}(a) \, y_1(a)$, to get $\tilde{y}_2(a)=y_2(a)$ (for each $a\in [0,\infty )$), obtaining

$$\begin{aligned} r_H(a)\mathop {=}\limits ^{{\mathrm {def}}}\frac{g_H(a)}{P(a)}=b'\left( u(a)\,e(m(a))\right) e(m(a)-\tilde{y}_1(a)\,e(m(a))-\tilde{y}_2(a) d'(u(a)) \end{aligned}$$

with

52 $$\begin{aligned} \dot{\tilde{y}}_1&=-(1-u)e'(m)\tilde{y}_1-\hat{P}\, b'(u\,e(m))\, u\, e'(m), \quad \mathop {\lim }\limits _{A\rightarrow \infty } \tilde{y}_1(A)=0, \nonumber \\ \dot{\tilde{y}}_2&=d(u)\tilde{y}_2-b(u\, e(m)), \quad \quad \mathop {\lim }\limits _{A\rightarrow \infty } \tilde{y}_2(A)=0, \end{aligned}$$

which is to be compared with

53 $$\begin{aligned} r(a)&=b'\left( u(a) e(m(a))\right) e(m(a))\nonumber \\&\quad -e\left( m(a)\right) \mathop {\int }\limits _{a}^{\infty }\hat{P}(\alpha )\,b'\left( u(\alpha ) e(m(\alpha ))\right) u(\alpha )e'(m(\alpha )) \hat{\varDelta }_{m}(\alpha ) \,\mathrm{d}\alpha \nonumber \\&\quad - d'(u(a))\mathop {\int }\limits _0^{\infty } \hat{P}(\alpha ) b \left( u(\alpha ) \, e(m(\alpha ))\right) \end{aligned}$$

with

54 $$\begin{aligned}&\hat{y}_1 \mathop {=}\limits ^{{\mathrm {def}}}\mathop {\int }\limits _a^{\infty } \hat{P}(\alpha )\, b'\left( u(\alpha ) e(m(\alpha ))\right) \, u\, e'(m(\alpha ))\hat{\varDelta }_{m}(\alpha )\, \mathrm{d}\alpha , \end{aligned}$$

55 $$\begin{aligned}&\hat{y}_2\mathop {=}\limits ^{{\mathrm {def}}}\mathop {\int }\limits _a^{\infty } \hat{P}(\alpha ) \, b\left( u(\alpha )\, e(m(\alpha ))\right) \, \mathrm{d}\alpha , \end{aligned}$$

where we now dropped the second argument of $\hat{P}$ and $\hat{\varDelta }_m$ on the understanding that $\hat{P}(a)=\hat{\varDelta }_m(a)=1$. The structure of the above expressions is

- for fitness returns:
  56 $$\begin{aligned} \hat{y}(a) = \mathop {\int }\limits _a^{\infty } {\phi }(\alpha ) z(\alpha ) \, \mathrm{d}\alpha , \quad \dot{x}=-\psi \,z, \quad z(a)=1 \end{aligned}$$
- for Pontryagin:
  57 $$\begin{aligned} \dot{\tilde{y}}=\psi \tilde{y} -\phi , \quad \mathop {\lim }\limits _{A\rightarrow \infty } \tilde{y}(A)=0. \end{aligned}$$

Expanding the integrals gives

58 $$\begin{aligned} \hat{y}(a)&=\mathop {\int }\limits _a^{\infty } \phi (\alpha ) \mathrm{exp}\left( -\mathop {\int }\limits _a^{\alpha } \psi (\tau )\, \mathrm{d}\tau \right) \mathrm{d}\alpha , \end{aligned}$$

59 $$\begin{aligned} \tilde{y}(a)&=-\mathop {\int }\limits _0^a \phi (\alpha ) \mathrm{exp} \left( \mathop {\int }\limits _{\alpha }^a \psi (\tau ) \mathrm{d}\tau \right) \, \mathrm{d}\alpha + \tilde{y}(0) \, \mathrm{exp} \left( \mathop {\int }\limits _0^a \psi (\tau )\, \mathrm{d}\tau \right) \end{aligned}$$

with

60 $$\begin{aligned} \tilde{y}(0)&=\mathrm{exp} \left( - \mathop {\int }\limits _0^{\infty } \psi (\tau )\mathrm{d}\tau \right) \mathop {\int }\limits _0^{\infty } \phi (\alpha ) \mathrm{exp} \left( \mathop {\int }\limits _{\alpha }^{\infty } \psi (\tau )\mathrm{d}\tau \right) \mathrm{d}\, \alpha \nonumber \\&=\mathop {\int }\limits _0^{\infty } \phi (\alpha ) \, \mathrm{exp}\left( \mathop {\int }\limits _0^{\alpha } \psi (\tau )\, \mathrm{d}\tau \right) \, \mathrm{d}\,{\alpha }. \end{aligned}$$

Hence,

61 $$\begin{aligned} \tilde{y}(a)&=- \mathop {\int }\limits _0^{a} \phi (\alpha ) \mathrm{exp} \left( \mathop {\int } \limits _{\alpha }^a \psi (\tau )\,\mathrm{d}\tau \right) \mathrm{d}\alpha +\mathop {\int }\limits _0^{\infty } \phi (\alpha ) \, \mathrm{exp} \left( \mathop {\int }_{\alpha }^{a} \psi (\tau )\, \mathrm{d}\tau \right) \, \mathrm{d} \alpha \nonumber \\&=\mathop {\int }\limits _a^{\infty } \phi (\alpha ) \, \mathrm{exp} \left( -\mathop {\int }_a^{\alpha } \psi (\tau )\, \mathrm{d}\tau \right) \,\mathrm{d} \alpha . \end{aligned}$$

Therefore, indeed $\tilde{y}_1=\hat{y}_1$ and $\tilde{y}_2=\hat{y}_2$.

## Appendix 5: Basic concepts of dynamic optimal control theory

One of the goals in this paper was to elucidate in an accessible manner the connection between the fitness returns of evolutionary ecology and Pontryagin’s maximum principle. This appendix considers the more general problem of linking the more intuitive, better known, and more general optimality principle of Bellman with Pontryagin’s maximum principle. To this end we derive the latter from the former. While both principles are commonly formulated for optimal strategies, we have reengineered the argument so that in the initial steps we consider just any fixed strategy in order to put in the limelight the direct conceptual link between fitness returns and the local variant of Pontryagin’s maximum principle.

### Appendix 5.1: The optimal control problem

Consider a dynamic system described by a state equation

62 $$\begin{aligned} \dot{x}(t)=f\left( x(t),u(t)\right) , \quad x(0)=x_0, \end{aligned}$$

where $x(t)\in {\mathbb {R}}^n$ is a state variable, $u(t)\in {\mathbb {R}}^m$ is a control variable, $f:{\mathbb {R}}^n\times {\mathbb {R}}^m \rightarrow {\mathbb {R}}^n$ is assumed to be continuously differentiable.

The control aim is to maximise the objective function:

63 $$\begin{aligned} J&=\mathop {\int }\limits _0^T\varPi \left( x(t),u(t)\right) \, \mathrm{d}t+ S\left( x(T)\right) , \end{aligned}$$

where $\varPi$ and *S* are assumed to be continuously differentiable.

The path *x*(*t*), $t\in [0,T]$, is called a state trajectory and *u*(*t*), $t\in [0,T]$, is called a control (decision, action).

Usually, the control variable *u*(*t*) is assumed to be piecewise continuous and constrained as follows:

64 $$\begin{aligned} u(t)\in \varOmega (t)\subset {\mathbb {R}}^m, \quad t\in [0,T]. \end{aligned}$$

If *u*(*t*) satisfies Condition (64) for each $t\in [0,T]$, we call *u* an admissible control.

The optimal control problem is to find an admissible control $u^*$ which maximises the objective function (63) subject to Constraint (62). Such a control $u^*$ is called an optimal control, the corresponding state trajectory is denoted by $x^*$ and is called the optimal trajectory under $u^*$, $J(u^*)$ or $J^*$ then denotes the optimal value of *J*.

### Appendix 5.2: Bellman’s optimality principle

Bellman’s optimality principle which has to be satisfied for an optimal control $u^*$ (Bellman 1957) reads: “an optimal policy has the property that whatever the initial state and initial decision are, the remaining decisions must constitute an optimal policy with regard to the state resulting from the first decision.”

Observing Fig. 2, we can state Bellman’s optimality principle as a proposition:

#### **Proposition**

If $a\,b\,e$ is the optimal path from *a* to *e*, then $b\, e$ is the optimal path from *b* to *e*.

#### *Proof*

Suppose it is not. Then there is another path (note that existence is assumed here) $b\,c\,e$ which is optimal from *b* to *e*, i.e. $J_{bce} > J_{be}$. But then $J_{abe} = J_{ab}+J_{be}< J_{ab}+ J_{bce}= J_{abce}$. This contradicts the hypothesis that $a\,b\,e$ is the optimal path from *a* to *e*. $\square$

### Appendix 5.3: The value function for any fixed *u*

We can formulate Bellman’s optimality principle using the so-called value function $V:{\mathbb {R}}^n \times {\mathbb {R}}\rightarrow {\mathbb {R}}$ for a given (but not necessarily optimal) control $u\in \varOmega$. This value function is defined as

65 $$\begin{aligned} V(x_t,t;u)\mathop {=}\limits ^{{\mathrm {def}}}\mathop {\int }\limits _t^T\varPi \left( x(s),u(s)\right) \, \mathrm{d}s + S\left( x(T)\right) , \end{aligned}$$

where for $$s\ge t$$

$$\begin{aligned} \frac{\mathrm{d}x}{\mathrm{d}s} = f \left( x, u\right) , \quad x(t)=x_t. \end{aligned}$$

In the original work of Bellman (1957) the value function was defined for an optimal strategy *u*, as the main focus of his work was finding this optimal strategy. We, however, formulate the value function for any *u* and introduce the Bellman’s optimality principle with *u* being optimal at the further step of this derivation.

In Fig. 3 we illustrate the value function in the (*x*, *t*)-space for a given *u*.

Note that incremental changes in *J* from *t* to $t+{\varDelta }_t$ are given by the integral of $\varPi (x,u)$ from *t* to $t+{\varDelta }_t$. Considering that the change in the objective function consists of the incremental changes in *J* from *t* to $t+{\varDelta }_t$ plus the value function $V(x+{\varDelta }_x, t+{\varDelta }_t;u)$ at time $t+{\varDelta }_t$ (for fixed *u*) we can write

66 $$\begin{aligned} V(x_t,t;u)&=\mathop {\int }\limits _t^{t+{\varDelta }_t}\varPi \left( x(\tau ),u(\tau )\right) \mathrm{d}\tau + V(x(t+{\varDelta }_t), t+{\varDelta }_t;u) . \end{aligned}$$

Since $\varPi$ is continuous in *t*, the integral in (66) can be approximated by $\varPi (x_t,u(t))\, {\varDelta }_t$, so that we can rewrite (66) as

67 $$\begin{aligned} V(x_t,t;u)=\varPi (x_t,u(t))\, {\varDelta }_t + V\left( x(t+{\varDelta }_t),t+{\varDelta }_t;u\right) +o({\varDelta }_t). \end{aligned}$$

If *V* is continuously differentiable, we can use the Taylor expansion of *V* with respect to ${\varDelta }_t$ to obtain

68 $$\begin{aligned} V(x(t+{\varDelta }_t), t+{\varDelta }_t;u)&=V(x_t, t;u)+\left( \frac{\partial \, V(x_t,t;u)}{\partial x}\dot{x}+\frac{\partial \, V(x_t,t;u)}{\partial t} \right) {\varDelta }_t \nonumber \\&\quad + o({\varDelta }_t). \end{aligned}$$

Substituting *x* from (62) we obtain

69 $$\begin{aligned} V(x_t,t;u)&= \varPi (x_t,u(t)){\varDelta }_t + V(x_t,t;u) + \frac{\partial \,V(x_t,t;u)}{\partial \,x} f(x_t,u(t)) {\varDelta }_t \nonumber \\&\quad +\frac{\partial \,V(x_t,t;u)}{\partial \,t}{\varDelta }_t + o({\varDelta }_t). \end{aligned}$$

By canceling $V(x_t,t;u)$ on both sides and then dividing by ${\varDelta }_t$ we get

70 $$\begin{aligned} 0=\varPi (x_t,u(t))+\frac{\partial \,V(x_t,t;u)}{\partial \,x} \,f(x_t,u(t))+\frac{\partial \,V(x_t,t;u)}{\partial \,t}+\frac{o({\varDelta }_t)}{{\varDelta }_t}. \end{aligned}$$

By letting ${\varDelta }_t \downarrow 0$, we obtain

71 $$\begin{aligned} 0=\varPi (x_t,u(t)) + \frac{\partial \,V(x_t,t;u)}{\partial \,x}\,f(x_t,u(t))+ \frac{\partial \,V(x_t,t;u)}{\partial \,t} \end{aligned}$$

with the boundary condition $V(x(T),T;u)=S\left( x(T)\right)$. Here $\frac{\partial \,V(x_t,t;u)}{\partial \,x}$ can be interpreted as the marginal contribution of the state variable *x* to the objective function for a fixed *u*. We denote it by $y(t)\in {\mathbb {R}}^n$ and call it the costate vector (also known as adjoint or auxiliary vector in optimisation and control theory and as a shadow price in economics), thus

72 $$\begin{aligned} y(t)\mathop {=}\limits ^{{\mathrm {def}}}\frac{\partial \,V(x_t,t;u)}{\partial \,x}, \end{aligned}$$

and introduce the so-called Hamiltonian^4^

73 $$\begin{aligned} H(x(t),u(t),y(t))\mathop {=}\limits ^{{\mathrm {def}}}\varPi (x(t),u(t))+(y(t))^T \, f(x(t),u(t)). \end{aligned}$$

Note that the previous definition for our example identifies *y* with the marginal fitness return per unit of change in the state variables at age *a* (here time *t*). Below we shall focus on optimal controls and trajectories, and in this way derive the equations for *y* customarily encountered in the literature on Pontryagins maximum principle.

### Appendix 5.4: From Bellman’s optimality principle to Pontryagin’s maximum principle

If *u* is optimal, i.e., if $u=u^*$, the following condition has to hold:

74 $$\begin{aligned} 0=\mathop {\max }\limits _{u(t)\in \varOmega (t)}\left\{ H\left( x_t,u(t),\frac{\partial \,V(x_t,t;u)}{\partial \,x}\right) +\frac{\partial \,V(x_t,t;u)}{\partial \,t}\right\} . \end{aligned}$$

Equation (74) is called the Hamilton–Jacobi–Bellman (HJB) equation (Bellman 1957) and gives necessary and sufficient conditions that an optimal *u* has to satisfy, if the value function is differentiable. From Eq. (74) we can get the Hamiltonian maximizing condition, or maximum principle, denoting the costate corresponding to $u^*$ as $y^*$ as

75 $$\begin{aligned} H(x^*(t),u^*(t),y^*(t))+\frac{\partial \, V(x^*(t),t;u^*)}{\partial \, t}&\ge H (x^*(t),\psi ,y^*(t))+\frac{\partial \, V(x^*(t),t;u^*)}{\partial \, t}, \end{aligned}$$

for all $\psi \in \varOmega (t),$ which leads to inequality

76 $$\begin{aligned} H(x^*(t),u^*(t),y^*(t)) \ge H (x^*(t),\psi ,y^*(t)). \end{aligned}$$

### Appendix 5.5: Derivation of the costate (adjoint) equation

We in this narrowed context return to the after effect of infinitesimal changes in the state variables. Let

77 $$\begin{aligned} x(t)=x^*(t)+{\varDelta }_x(t) \end{aligned}$$

where ${\varDelta }_x(t)$, $\Vert {\varDelta }_x(t)\Vert \downarrow 0$. As $x^*$ is the optimal state trajectory, necessarily

78 $$\begin{aligned}&H\left( x^*(t),u^*(t),\frac{\partial \, V(x^*(t),t;u^*)}{\partial \, x}\right) +\frac{\partial \, V(x^*(t),t;u^*)}{\partial \, t} \nonumber \\&\quad \ge H \left( x(t),u^*(t),\frac{\partial \, V(x(t),t;u^*)}{\partial \, x}\right) +\frac{\partial \, V(x(t),t;u^*)}{\partial \, t}. \end{aligned}$$

The left hand side of Inequality (78) equals zero, since $u^*$ is the optimal control. In general, the right hand side is less or equal to zero and would be zero for $x=x^*$. Since *x*(*t*) is unconstrained, the partial derivative of the right hand side of Inequality (78) with respect to *x* has to be equal to zero for $x=x^*$, i.e.,

79 $$\begin{aligned} \left[ \frac{\partial H\left( x_t,u^*(t), \frac{\partial V(x_t,t;u^*)}{\partial x},t\right) }{\partial x}+\frac{\partial ^2 V (x_t,t)}{\partial t \,\partial x}\right] _{x=x^*}=0. \end{aligned}$$

(Note the implicit assumption that *V* is twice continuously differentiable.) By definition of the Hamiltonian, at $u=u^*$ and $x=x^*$,

80 $$\begin{aligned} {\frac{\partial \varPi }{\partial x}}+ {\frac{\partial V}{\partial x}} {\frac{\partial f}{\partial x}}+ f^T {\frac{\partial ^2 V}{\partial x^2}} + {\frac{\partial ^2 V}{\partial t \partial x}} ={\frac{\partial \varPi }{\partial x}}+ {\frac{\partial V}{\partial x}} {\frac{\partial f}{\partial x}}+ \left( \frac{\partial ^2 V}{\partial x^2}\,f\right) ^T + \frac{\partial ^2 V}{\partial t \partial x}=0. \end{aligned}$$

By definition (72) of *y*(*t*)

81 $$\begin{aligned} \frac{\mathrm{d} \left( \frac{\partial V}{\partial x}\right) }{\mathrm{d}t}&=\left( \frac{\mathrm{d} \left( \frac{\partial V}{\partial x_1}\right) }{\mathrm{d}t}, \ldots \frac{\mathrm{d} \left( \frac{\partial V}{\partial x_n}\right) }{\mathrm{d}t} \right) \nonumber \\&=\left( \frac{\partial ^2\, V}{\partial \, x^2}\, \dot{x}\right) ^T + \frac{\partial ^2\, V }{\partial \,x \partial t}= \left( \frac{\partial ^2\, V}{\partial \, x^2}\, f\right) ^T + \frac{\partial ^2\, V }{\partial \,x \partial t}. \end{aligned}$$

From Formulas (80) and (81) we have

82 $$\begin{aligned} \frac{\mathrm{d}{\left( \frac{\partial \, V}{\partial x}\right) }}{\mathrm{d}t}= - \frac{\partial \varPi }{\partial x}- \frac{\partial V}{\partial x}\, \frac{\partial \, f}{\partial \, x}. \end{aligned}$$

Substituting the costate from Formula (72), we obtain

$$\begin{aligned} \dot{y}=-\frac{\partial \, \varPi }{\partial \, x}-y \, \frac{\partial \, f}{\partial \, x}. \end{aligned}$$

Substituting the Hamiltonian (73) into this expression, we obtain

83 $$\begin{aligned} \dot{y}=-\frac{\partial \, H}{\partial \, x}. \end{aligned}$$

Terminal boundary (or transversality) conditions are defined as

84 $$\begin{aligned} y(T)=\frac{\partial \, S(x(T))}{\partial \, x}. \end{aligned}$$

Equations (83) and (84) determine the adjoint variables From Eq. (73) we can rewrite the state equation as

85 $$\begin{aligned} \dot{x}=f=\frac{\partial \, H}{\partial \, y}. \end{aligned}$$

Combining Formulas (83), (84), (85), and (62) we get

86 $$\begin{aligned} \left\{ \begin{array}{l} \dot{x}=\frac{\partial \, H}{\partial \, y}, \quad x(0)=x_0, \\ \dot{y}=-\frac{\partial \, H}{\partial \, x}, \quad y(T)=\frac{\partial S(x(T))}{\partial x}. \end{array}\right. \end{aligned}$$

Equation (86) is called a canonical system of equations or canonical adjoints.

### Appendix 5.6: Pontryagin’s maximum principle

The necessary conditions for $u^*$ to be an optimal control are:

87 $$\begin{aligned} \left\{ \begin{array}{l} \dot{x}^* = f(x^*,u^*,t),\quad x^*(0)=x_0,\\ \dot{y}=-\frac{\partial \, H (x^*,u^*,y)}{\partial \, x}, \quad y(T) = \frac{\partial \, S(x^*(T))}{\partial \, x},\\ H\left( x^*(t),u^*(t),y(t)\right) \ge H\left( x^*(t),u,y(t)\right) , \end{array} \right. \end{aligned}$$

for all $u\in \varOmega (t)$, $t\in [0,T]$, where the adjoint (costate) variables *y*(*t*) now correspond to the sensitivities of the value function for the optimal *u* to a state change at *t*.

## Footnotes

1 Here star denotes optimality.

2 Using integration by parts, ${\mathbf {E}} (\lambda )=-\mathop {\int }\nolimits _0^{\infty }\dot{P} (\alpha ;u) \,\lambda (\alpha ;u) \mathrm{d}\alpha = P(0)\lambda (0)-\mathop {\lim }\nolimits _{T\rightarrow \infty }P(T) \mathop {\lim }\nolimits _{T\rightarrow \infty }\lambda (T) + R_0=R_0$, because $P(0)\lambda (0)=\mathop {\lim }\nolimits _{T\rightarrow \infty }P(T) \mathop {\lim }\nolimits _{T\rightarrow \infty }\lambda (T)=0$.

3 Let $\underline{m}$ be the lifetime offspring number. Then $1=R_0\mathop {=}\limits ^{{\mathrm {def}}}{\mathbf {E}} (\underline{m})={\mathbf {E}} (\underline{m}|\underline{a})= {\mathbf {E}}(\lambda (\underline{a}))$. Since for a Poisson random variable with mean $\lambda$ its variance also equals $\lambda$, $\sigma ^2=\mathrm{Var} (\underline{m}) = {\mathbf {E}} (\underline{m}^2) - ({\mathbf {E}} (\underline{m}))^2={\mathbf {E}} ({\mathbf {E}}(\underline{m}^2|\underline{a})) - 1= {\mathbf {E}} (\mathrm{Var} (\underline{m}|\underline{a}) + ({\mathbf {E}} (\underline{m}|\underline{a}))^2) - 1={\mathbf {E}} (\lambda (\underline{a}))+{\mathbf {E}} (\lambda ^2(\underline{a}))-1={\mathbf {E}} (\lambda ^2(\underline{a}))={\mathbf {E}} (\underline{\lambda }^2).$
